# A new model of Hopfield network with fractional-order neurons for parameter estimation

**DOI:** 10.1007/s11071-021-06398-z

**Published:** 2021-04-05

**Authors:** Stefano Fazzino, Riccardo Caponetto, Luca Patanè

**Affiliations:** 1 Dipartimento di Ingegneria Elettrica Elettronica e Informatica, Università degli Studi di Catania, Viale Andrea Doria 6, 95125 Catania, Italy; 2 Dipartimento di Ingegneria, Università degli Studi di Messina, Contrada di Dio, 98166 Messina, Italy

**Keywords:** Hopfield network, Fractional-order, System identification, Adomian decomposition

## Abstract

In this work, we study an application of fractional-order Hopfield neural networks for optimization problem solving. The proposed network was simulated using a semi-analytical method based on Adomian decomposition,, and it was applied to the on-line estimation of time-varying parameters of nonlinear dynamical systems. Through simulations, it was demonstrated how fractional-order neurons influence the convergence of the Hopfield network, improving the performance of the parameter identification process if compared with integer-order implementations. Two different approaches for computing fractional derivatives were considered and compared as a function of the fractional-order of the derivatives: the Caputo and the Caputo–Fabrizio definitions. Simulation results related to different benchmarks commonly adopted in the literature are reported to demonstrate the suitability of the proposed architecture in the field of on-line parameter estimation.

## Introduction

The problem of system identification is ubiquitous in different research fields ranging from biology to engineering applications. The access to mathematical models of the considered phenomena, often in the form of systems of nonlinear differential equations, can help the identification process exploiting the available apriori knowledge. However, the parameters of any models present inaccuracies that need to be corrected on the basis of the experimental data available (i.e. grey-box modelling) [27]. The parameter identification problem can be solved using different techniques based on least square and maximum likelihood methods even if other techniques based on genetic algorithms, neural networks, and neural-fuzzy systems are also adopted [26, 40, 41].

It is well established that Hopfield neural networks (HNNs) [17, 18] can be used to solve optimization problems, including parameter estimation in the context of system identification [9]. Interesting examples are reported in [6, 20, 21, 39] where Hopfield neural networks were applied for on-line identification of grey-box models, to continuously obtain an estimation of the system parameters.

The application of Hopfield models to optimization is a consequence of study its stability with an energy function method: the network seeks a minimum of its Lyapunov function which is built from the target function. In parameters identification problems, Hopfield network is designed in a way that its Lyapunov function coincides with the prediction error so that the network evolution approaches a minimum of the error. In [8] and [7], the authors presented a stability analysis of HNNs for on-line parameters identification, which are different from conventional HNNs because weights and biases are time-variant and depend on the state variables of the modelled system. A comprehensive study of the problem of using HNNs for on-line parameter estimation has been provided in [4]. While these studies are based on integer order real neuron models, in our work we examine a generalization of HNNs which dynamic can be described by fractional-order differential equations [10] and, for the first time, we apply them to parameter estimation problems. Fractional-order systems were recently applied to improve the accuracy of epidemic phenomena and electrical models [1, 11] and also to realize more precise and robust control systems [31, 35]. In [12] a fractional control protocol has been applied to multi-agent systems to enhance the convergence speed and robustness of the system under constant disturbances. A new variable fractional-order derivative, applied to the coronavirus epidemic phenomena, has been proposed in [38] where, by using the fixed point theory, the existence and uniqueness of the solution have been demonstrated. In [36] a novel fractional-order PID sliding mode controller with neural network observer is proposed and applied to hypersonic vehicles. Another application of fractional-order PID controllers has been presented in [16] where a particle swarm optimization algorithm is used to search for the optimal parameters of the controllers. The interest of the research community in the field of fractional-order HNN is further demonstrated by recent theoretical analyses. Global stability problem for fractional-order HNN has been investigated in [42], adopting an intermittent control. Moreover, a new three-dimensional fractional-order HNN with a delay has been investigated, proposing a synchronization method based on a state observer [19]. The role of activation functions has been also deepened in [33] where the stability and synchronization of fractional-order HNN are analysed using Lyapunov functions. In [14] classical and non-integer model order reduction methodologies have been presented demonstrating the suitability of fractional calculus in compressing information while modelling systems and in describing long-term memory effects.

In on-line applications the convergence time is particularly relevant, therefore, its relation with fractional-order value has been investigated in simulations which have been carried out using Adomian algorithm, a semi-analytic method for simulating fractional-order differential equations [3]. Different fractional derivative definitions are available in the literature [5, 33, 34]. The Caputo and the Caputo–Fabrizio definitions were applied to develop the proposed fractional-order HNN and compared on two different cases of study commonly adopted in the literature. The former is related to the estimation of the parameter in the well-known Lorenz system that exhibits a chaotic behaviour [28]. Besides the complexity of the system dynamics, the approach can be easily applied to the system that is linear referring to the parameters. It has been used as testbed also by Lazzs and coauthors in [26] to evaluate the performances of parameter estimation methods based on swarm intelligence. Furthermore, the latter case of study is related to a mechanical two-cart system, adopted in [4] to evaluate the identification performance of an integer-order HNN. The role of the fractional order of the derivatives was investigated to demonstrate the improvements introduced by the proposed architecture when compared with traditional integer-order solutions.

The main contribution of this work is summarized as follow:

1. An on-line identification method for grey-box models, based on an HNN, mathematically formulated in the context of fractional-order systems is proposed.
2. The Caputo and Caputo–Fabrizio definitions of the fractional-order derivative are considered and compared in the field of parameter identification.
3. The Adomian decomposition method is applied to guarantee an accurate approximation of the fractional derivatives and a fast convergence of the optimization procedure.
4. The performance of the proposed solutions, both in terms of convergence time and prediction accuracy, are reported and compared, using well-established benchmarks, with other techniques adopted in the literature.
5. The relation between the obtained performance and the fractional-order of the derivatives is deeply investigated to better understand the advantages and bottlenecks of the proposed approach.

The remainder of this paper is organized as follows: Sect. 2 describes the proposed HNN; Sect. 3 presents the Adomian decomposition method which has been used in simulations; Sect. 4 deals with the application of fractional-order Hopfield networks to time-varying parameter identification; and Sect. 5 describes the application of our method and main findings. Finally, conclusions are drawn in Sect. 6.

## Fractional Hopfield neural network

A generalization of the HNN model is represented by the introduction of a fractional-order neuron. Fractional calculus has been suggested as an appropriate mathematical tool to describe a wide variety of physical, chemical and biological processes and, in particular, those following the so-called *power law*. Further, fractional calculus is characterized by long-term memory and non-locality: fractional derivatives of a function depend not only on local conditions of the evaluated time but also on all the history of the function [37]. A theoretical study of the behaviour and stability of fractional-order Hopfield networks (FOHNN) was presented in [25], while implementation in the form of an analog circuit in [32].

For our work, we consider FOHNN based on real-valued neurons. The network has a recurrent structure with all-to-all interconnections composed by *N* real-valued neurons. The state of the $n^{th}$ neuron is denoted by a real $s_{n}(t)$ variable for $n=1,\ldots ,N$. Each neuron has bias input $\varvec{I}=\{I_j\}$ and is connected to every other neuron through weights $\varvec{W} = \{w_{jk}\}$, where $w_{jk}\in \mathbb {R}$ is the weight connecting $j^{th}$ and $k^{th}$ neuron. Each neuron receives inputs from all other neurons, performs a weighted sum of the inputs $\varvec{\xi }$ and passes the sum through the following activation function:

1 $$\begin{aligned} F(x)=\frac{1}{2}\left( 1+\mathrm {tanh}\left( \frac{x}{\chi }\right) \right) \end{aligned}$$

where $\chi$ determines the slope of the activation function.

Dynamical model of the network can be described in vectorial form by the following fractional-order differential equation:

2 $$\begin{aligned} _{_{0}}D_{t}^{(\alpha )}\varvec{\xi }(t) = \varvec{W}F(\varvec{\xi }(t))-\varvec{I} \end{aligned}$$

where $_{_{0}}D_{t}^{(\alpha )}$ is the fractional-order derivative of order $\alpha$,

3 $$\begin{aligned} \varvec{\xi }(t)=[\xi _1(t),\ldots ,\xi _N(t)]^{T} \end{aligned}$$

are the input potentials to neurons, *F* is the activation function defined in Eq. (1) and $\varvec{I}$ is the bias vector. The state vector of the network is expressed as $\varvec{s}=F(\varvec{\xi }(t))$.

Equation (2) has the same structure of Abe formulation of Hopfield neuron which is widely used in optimization problems [2]. Several definitions of fractional-order time derivative are available in the literature. In this work, the Caputo and the Caputo–Fabrizio definitions have been taken into account [15].

A sufficient condition for the stability of the dynamics is that the matrix of synaptic weights $\varvec{W}$ is symmetric with non-negative diagonal entries, that is, $\varvec{W}=\varvec{W}^T$, $w_{ii}\ge 0$ [23].

The Lyapunov or energy function of the state $\varvec{s}$ is:

4 $$\begin{aligned} E=-\frac{1}{2}\varvec{s}^{T}\varvec{W}\varvec{s}+\varvec{s}^{T}\varvec{I} \end{aligned}$$

The existence and the specific characteristics of this Lyapunov function guarantee that the network evolves spontaneously in the descending direction of such a function until approaching the minima of the energy function.

## Adomian decomposition method

Finding numerical solutions to fractional differential equations can be computationally intensive due to the effect of non-local derivatives in which all previous time points contribute to the current iteration [30].

However, a high-accurate approximation of fractional derivatives which demonstrates fast convergence to the solution can be obtained from the Adomian decomposition method which has been developed by George Adomian [3]. The algorithm is based on a decomposition of the nonlinear operator as a series where each term is a generalized polynomial called Adomian polynomial.

Following the notation introduced in [13], we consider the equation

5 $$\begin{aligned} \varvec{F}(\varvec{x}(t))=\varvec{g}(t) \end{aligned}$$

with

6 $$\begin{aligned} \varvec{x}(t)=(x_1(t), x_2(t),...,x_n(t))^T \end{aligned}$$

and

7 $$\begin{aligned} \varvec{g}(t)=(g_1(t),g_2(t),...,g_n(t))^T \end{aligned}$$

where $\varvec{F}$ represents a nonlinear ordinary differential operator involving both linear and nonlinear terms, and $\mathbf {g}(t)$ is an inhomogeneous term.

The Adomian decomposition method requires that $\varvec{F}$ is separated into three terms $\varvec{F}=\varvec{L}+\varvec{R}+\varvec{N}$, where the differential operator $\varvec{L}$ may be considered as the highest order derivative in the equation, $\varvec{R}$ is the remainder of the differential operator and $\varvec{N}$ expresses the nonlinear terms.

Consequently, the system in Eq. (5) becomes

8 $$\begin{aligned} \varvec{L}(\varvec{x})+\varvec{R}(\varvec{x})+\varvec{N}(\varvec{x})=\varvec{g}(t) \end{aligned}$$

Here, $\varvec{L}$ is chosen to be easily invertible and applying the inverse operator $\varvec{L^{-1}}$ to both sides of Eq. (8) gives

9 $$\begin{aligned} \varvec{x}(t)=\varvec{\varPsi }_0+\varvec{L^{-1}}(\varvec{g}(t))-\varvec{L^{-1}}(\varvec{R}(\varvec{x}))-\varvec{L^{-1}}(\varvec{N}(\varvec{x})) \end{aligned}$$

where $\varvec{\varPsi }_0$ is the kernel of the operator $\varvec{L^{-1}}$.

The Adomian decomposition method admits the decomposition of $\varvec{x}$ into an infinite series of components

10 $$\begin{aligned} \varvec{x}(t)=\sum _{i=0}^{\infty }\varvec{x}^{(i)}=\sum _{i=0}^{\infty } \left( \begin{array}{c} x_1^{(i)} \\ x_2^{(i)} \\ \vdots \\ x_n^{(i)} \end{array} \right) \end{aligned}$$

and the nonlinear term $N(\varvec{x})$ into an infinite series of polynomials

11 $$\begin{aligned} \varvec{N}(\varvec{x})=\sum _{i=0}^{\infty }\varvec{A}^{(i)}=\sum _{i=0}^{\infty }\left( \begin{array}{c}A_1^{(i)} \\ A_2^{(i)} \\ \vdots \\ A_n^{(i)}\end{array}\right) \end{aligned}$$

where the components $A_j^{(i)}$ are called the Adomian polynomials which can be calculated by using the following expression

12 $$\begin{aligned} A_j^{(i)}=\frac{1}{i!}\left[ \frac{d^i}{d\lambda ^i} N_j\left( \sum _{k=0}^{i}\lambda ^k \varvec{x}^{(k)}\right) \right] _{\lambda =0} \end{aligned}$$

with $$i=0,\dots ,M-1$$ and $$j=1,\dots ,n$$.

Substituting (10) and (11) into Eq. (9) gives

13 $$\begin{aligned} \sum _{i=0}^{\infty }\varvec{x}^{(i)}= & {} \varvec{\varPsi }_0+\varvec{L^{-1}}(\varvec{g}(t))+\nonumber \\&-\varvec{L^{-1}}\left( \varvec{R}\left( \sum _{i=0}^{\infty }\varvec{x}^{(i)}\right) \right) \nonumber \\&- \varvec{L^{-1}}\left( \sum _{i=0}^{\infty }\varvec{A}^{(i)}\right) \end{aligned}$$

The components $\varvec{x}^i$ of the solution (10) can be easily calculated by using the recursive relation

14 $$\begin{aligned} \begin{aligned} \varvec{x}^{(0)}&=\varvec{\varPsi }_0+\varvec{L^{-1}}(\varvec{g}(t)) \\ \varvec{x}^{(1)}&=-\varvec{L^{-1}}(\varvec{R}(\varvec{x}^{(0)}))-\varvec{L^{-1}}(\varvec{A}^{(0)}) \\ \varvec{x}^{(2)}&=-\varvec{L^{-1}}(\varvec{R}(\varvec{x}^{(1)}))-\varvec{L^{-1}}(\varvec{A}^{(1)}) \\ \dots \\ \varvec{x}^{(k+1)}&=-\varvec{L^{-1}}(\varvec{R}(\varvec{x}^{(k)}))-\varvec{L^{-1}}(\varvec{A}^{(k)}), k\ge 0 \end{aligned} \end{aligned}$$

Having determined the first $M$ components $\varvec{x}^{(i)}$ of the solution, the $M$-term approximate solution in the interval $[t_0,t]$ can be defined as

15 $$\begin{aligned} \varvec{\tilde{x}}(t)=\sum _{i=0}^{M-1}\varvec{x}^{(i)} \end{aligned}$$

In order to calculate an approximate analytical solution to system of differential equations with Caputo’s derivative, we can consider $\mathbf{L} ^{-1}=\mathbf{J} ^{\alpha }_{t_0}$, where $\mathbf{J} ^{\alpha }_{t_0}$ is the Riemann–Liouville fractional integration of order $\alpha$, defined as

16 $$\begin{aligned} \mathbf{J} ^{\alpha }_{t_0}\varvec{f}(t)=\frac{1}{\varGamma (\alpha )}\int _{t_0}^t\frac{\varvec{f}(\tau )}{(t-\tau )^{1-\alpha }}\mathrm{d}\tau . \end{aligned}$$

As the Caputo’s fractional derivative is defined as

17 $$\begin{aligned} \mathbf{D} ^{\alpha }_{t_0}\varvec{f}(t)= & {} \mathbf{J} ^{m-\alpha }_{t_0}\left[ \frac{d^m}{dt^m}\varvec{f}(t)\right] \nonumber \\= & {} \frac{1}{\varGamma (m-\alpha )}\int _{t_0}^t\frac{\varvec{f}^{(m)}(\tau )}{(t-\tau )^{\alpha -m+1}}\mathrm{d}\tau \end{aligned}$$

where $m-1<\alpha \le m$ and $m \in \mathbb {N}$, by combining Eqs. (16) and (17) we obtain

18 $$\begin{aligned} \mathbf{J} ^{\alpha }_{t_0}{} \mathbf{D} ^{\alpha }_{t_0}\varvec{f}(t)=\varvec{f}(t)-\sum _{k=0}^{m-1}\frac{\varvec{f}^{(k)}(t_0)}{k!}(t-t_0)^k. \end{aligned}$$

In the same way, we can consider the Caputo–Fabrizio fractional-order derivative [29, 34]:

19 $$\begin{aligned}&^\text {FC}{} \mathbf{D} ^{\alpha }_{0}\varvec{f}(t)=\frac{(2-\alpha )M(\alpha )}{2(1-\alpha )}\int _{0}^t\nonumber \\&\quad \text {exp}\left( -\frac{\alpha }{1-\alpha }(t-\tau )\right) \varvec{f}^{(1)}(\tau )\mathrm{d}\tau \end{aligned}$$

given $t>0$ and $M(\alpha )$ a normalization constant depending on $\alpha$. In this case, the associated fractional-order integral is:

20 $$\begin{aligned} \mathbf{L} ^{-1}= & {} {^\text {FC}{} \mathbf{J} ^{\alpha }_{0}}\varvec{f}(t)=\frac{2(1-\alpha )}{(2-\alpha )M(\alpha )}(\varvec{f}(t)-\varvec{f}(0))\nonumber \\&+\frac{2\alpha }{(2-\alpha )M(\alpha )}\int _{0}^t\varvec{f}(\tau )\mathrm{d}\tau . \end{aligned}$$

In our experiments, we imposed:

21 $$\begin{aligned} M(\alpha )=\frac{2}{2-\alpha }. \end{aligned}$$

While numerical methods generally rely on discretization techniques of nonlinearities in equations and permit to calculate an approximate solution for specific values of times and require computer-intensive calculations, an analytical method like the Adomian’s gives a continuous approximation of unknown solution in terms of a truncated series (see Eq. 15) in which the original nonlinearity is transformed to other nonlinear terms (i.e. Adomian polynomials) [24].

## Parameter estimation using Hopfield networks

**Fig. 1:**
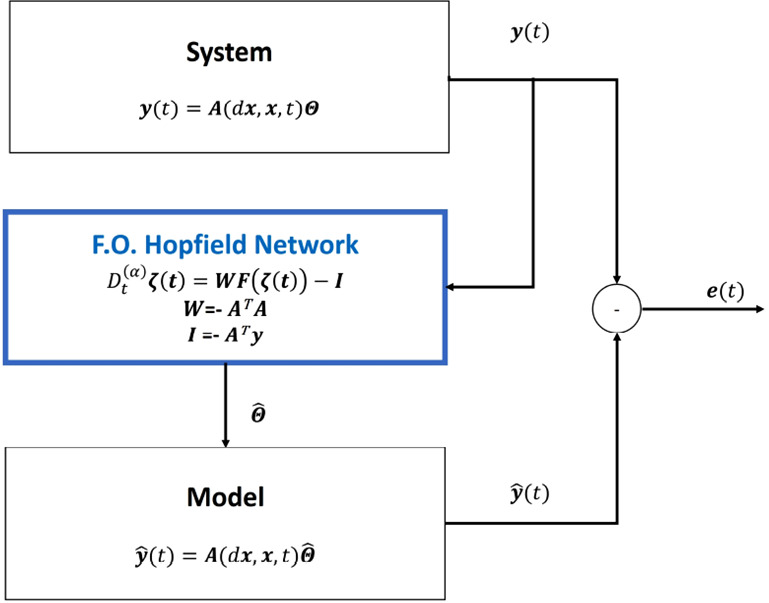
Neural network identification scheme. The parameters of the system are estimated using a FOHNN on the basis of the system matrix $\varvec{A}$ and using the output signal $\varvec{y(t)}$

The parameter estimation problem is the identification of the numeric value of uncertain, unknown or time-varying parameters when the ordinary differential equations (ODEs) of the model are known. This kind of optimization problem can be addressed by using Hopfield Networks, as described in [8] and [22]. A scheme of the proposed identification process is reported in Fig. 1.

In particular, the dynamical system is required to be linear in parameters (LIP); therefore, it can be expressed as follows:

22 $$\begin{aligned} \varvec{y}=\varvec{A}(\varvec{x},\varvec{u})\varvec{\theta } \end{aligned}$$

where $\varvec{y}$ is called output vector (that not necessarily corresponds to the physical output of the system), $\varvec{x}$ state vector, $\varvec{u}$ input vector and $\varvec{\theta }$ parameter vector and $\varvec{A}(\varvec{x},\varvec{u})$ is a matrix whose components are nonlinear functions of the state variables and inputs. It is also required that both $\varvec{y}$ and $\varvec{A}$ are measurable or known.

Once the system has been described in the LIP form, the problem of parameter estimation at each time step is equivalent to find the parameter values of $\varvec{\theta }$ that minimize the prediction error $\varvec{e}$ of the system, which is given by the difference between $\varvec{y}$ (the actual, measured value of the output) and $\varvec{\hat{y}}$ the output value that is calculated by substituting the estimated parameters $\varvec{\hat{\theta }}$ into the model:

23 $$\begin{aligned} \varvec{e}=\varvec{y}-\varvec{\hat{y}}=\varvec{y}-\varvec{A}(\varvec{x},\varvec{u})\varvec{\hat{\theta }} \end{aligned}$$

The target function is the squared norm of the prediction error:

24 $$\begin{aligned} E=\frac{1}{2}\left\Vert \varvec{e}\right\Vert ^{2}=\frac{1}{2}\varvec{e}^{T}\varvec{e} \end{aligned}$$

and, by using equation (23), it can be expressed as:

25 $$\begin{aligned} E=\frac{1}{2}\varvec{\hat{\theta }}^{T}\varvec{A}^{T}\varvec{A}\varvec{\hat{\theta }}-\varvec{\hat{\theta }}^{T}\varvec{A}^{T}\varvec{y}+\frac{1}{2}\left\Vert \varvec{y}\right\Vert ^{2} \end{aligned}$$

The last term of Eq. (25) can be neglected as it does not depend on the estimated parameters. Finally, we obtain the following energy function:

26 $$\begin{aligned} E=\frac{1}{2}\varvec{\hat{\theta }}^{T}\varvec{A}^{T}\varvec{A}\varvec{\hat{\theta }}-\varvec{\hat{\theta }}^{T}\varvec{A}^{T}\varvec{y} \end{aligned}$$

On the basis of the considerations reported in Sect. 2 and, following the Lyapunov function for HNN derived in Eq. (4), we can extract from Eq. (26) the following weights and biases:

27 $$\begin{aligned} \varvec{W}=-\varvec{A}^{T}\varvec{A} \qquad \varvec{I}=-\varvec{A}^{T}\varvec{y} \end{aligned}$$

As a consequence, the network defined by weights and biases in Eq. (27) has one neuron for each parameter to be estimated and $\varvec{\hat{\theta }}$ is the state that minimizes the energy function of the network.

Furthermore, as the activation function of the network (see Eq. (1)) has a limited range of variability, it is necessary to have previous knowledge of the maximum range of variability of parameters.

## Numerical simulations

In order to test the reliability of our model, a simulation tool based on the Adomian decomposition method was developed in ***Mathematica*** and tested to address some parameter estimation problems. All simulations were carried out with $M=8$ terms of Adomian polynomials as a good compromise between computational effort and numerical accuracy. Two cases of study, commonly adopted as a testbed for on-line parameter estimation technique, are considered: the chaotic Lorenz system and a mechanical two-cart system. We have analysed, in both cases, the effect of the fractional parameter $\alpha$ in a wide range, $\alpha \in [0.05,\; 1.5]$, evaluating the performance of the Caputo and Caputo–Fabrizio fractional derivative formulations.

### Parameter estimation of a Lorenz system

Parameter estimation in chaotic systems is an important topic in signal processing and control system theory [26]. A representative case of study is here reported applying the proposed identification strategy to the Lorenz oscillator. It is a three-dimensional dynamical system that exhibits chaotic flow and was named after Edward N. Lorenz, who derived it from the simplified equations of convection rolls in the atmosphere [28].

**Fig. 2:**
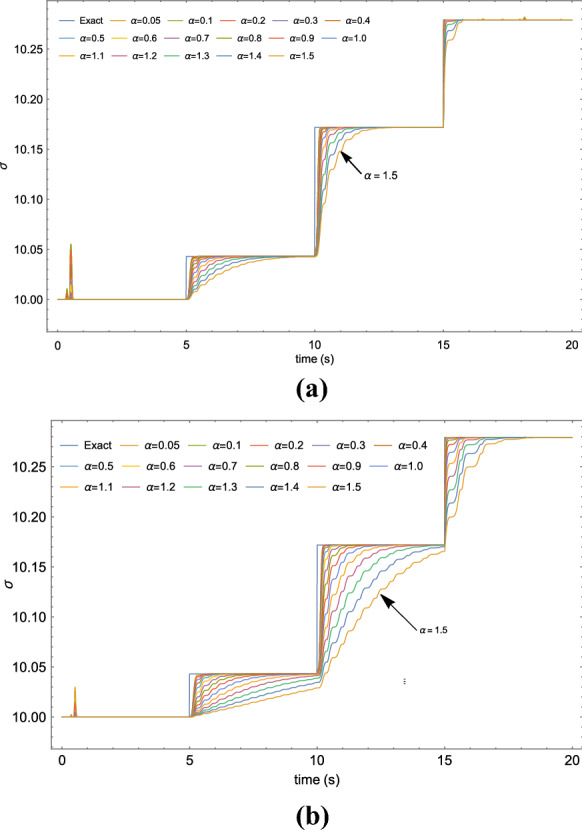
Estimation of $\sigma$ parameter of the Lorenz system at different fractional-order values adopting: **a** the Caputo–Fabrizio and **b** the Caputo derivative

**Table 1:** The precision of $\sigma$ estimation in Lorenz system with Caputo–Fabrizio (C–F) and Caputo (C) derivatives

Mean Squared Error				
$\alpha$	Average (C-F)	Average(C)	Std Deviation (C-F)	Std Deviation (C)
0.05	$1.03\times 10^{-4}$	$1.05\times 10^{-4}$	$1.05\times 10^{-3}$	$1.10 \times 10^{-3}$
0.1	$1.03\times 10^{-4}$	$1.10\times 10^{-4}$	$1.05\times 10^{-3}$	$1.14 \times 10^{-3}$
0.2	$1.03\times 10^{-4}$	$1.12\times 10^{-4}$	$1.07\times 10^{-3}$	$1.15 \times 10^{-3}$
0.3	$1.04\times 10^{-4}$	$1.12\times 10^{-4}$	$1.09\times 10^{-3}$	$1.19 \times 10^{-3}$
0.4	$1.04\times 10^{-4}$	$1.31\times 10^{-4}$	$1.09\times 10^{-3}$	$1.24 \times 10^{-3}$
0.5	$1.09\times 10^{-4}$	$1.45\times 10^{-4}$	$1.13\times 10^{-3}$	$1.29 \times 10^{-3}$
0.6	$1.10\times 10^{-4}$	$1.62\times 10^{-4}$	$1.14\times 10^{-3}$	$1.35 \times 10^{-3}$
0.7	$1.16\times 10^{-4}$	$1.86\times 10^{-4}$	$1.17\times 10^{-3}$	$1.41 \times 10^{-3}$
0.8	$1.26\times 10^{-4}$	$2.19\times 10^{-4}$	$1.22\times 10^{-3}$	$1.49 \times 10^{-3}$
0.9	$1.38\times 10^{-4}$	$3.39\times 10^{-4}$	$1.27\times 10^{-3}$	$1.58 \times 10^{-3}$
1.0	$1.53\times 10^{-4}$	$4.45\times 10^{-4}$	$1.32\times 10^{-3}$	$1.70 \times 10^{-3}$
1.1	$1.74\times 10^{-4}$	$6.03\times 10^{-4}$	$1.38\times 10^{-3}$	$1.86 \times 10^{-3}$
1.2	$2.02\times 10^{-4}$	$8.36\times 10^{-4}$	$1.45\times 10^{-3}$	$2.10 \times 10^{-3}$
1.3	$2.43\times 10^{-4}$	$1.18\times 10^{-3}$	$1.53\times 10^{-3}$	$2.45 \times 10^{-3}$
1.4	$3.02\times 10^{-4}$	$1.68\times 10^{-3}$	$1.64\times 10^{-3}$	$2.95 \times 10^{-3}$
1.5	$3.89\times 10^{-4}$	$1.68\times 10^{-3}$	$1.78\times 10^{-3}$	$3.61 \times 10^{-3}$

For the first time, he used the term “butterfly effect” to indicate the sensitive dependence on initial conditions: small variations of the initial condition in chaotic system may produce large variations in the long term behaviour.

Lorenz’s system can be described as:

28 $$\begin{aligned} \frac{dx_1(t)}{dt}&=\sigma (x_2(t)-x_1(t)) \nonumber \\ \frac{dx_2(t)}{dt}&=x_1(t)(\rho -x_3(t))-x_2(t) \nonumber \\ \frac{dx_3(t)}{dt}&=x_1(t)x_2(t)-\beta x_3(t) \end{aligned}$$

where $\sigma$ is called the Prandtl number and $\rho$ is called the Rayleigh number.

All parameters$\sigma , \rho ,\beta > 0$, but usually $\sigma =10$, $\beta =8/3$, while the system exhibits chaotic behaviour for $\rho = 28$.

Equation (28) are linear in parameters and can be written in the form $\varvec{y}=\varvec{A}(\varvec{x},\varvec{u})\varvec{\theta }$, where

29 $$\begin{aligned} \varvec{y}&=\begin{pmatrix}\frac{dx_1(t)}{dt} \\ \frac{dx_2(t)}{dt}+x_1(t)x_3(t)+x_2(t) \\ \frac{dx_3(t)}{dt}-x_1(t)x_2(t) \end{pmatrix}\nonumber \\ \varvec{A}&=\begin{pmatrix}x_2(t)-x_1(t) &{} 0 &{} 0 \\ 0 &{} x_1(t) &{} 0 \\ 0 &{} 0 &{} -x_3(t)\end{pmatrix} \nonumber \\ \varvec{\theta }&= \begin{pmatrix}\sigma \\ \rho \\ \beta \end{pmatrix} \end{aligned}$$

The system described in Eq. (28) has been simulated for 20 s with $\beta =8/3$, $\rho =28$ and considering an integration time step $\tau =0.1$, while $\sigma$ is randomly changed at every 50 s. At each time step $\tau$, the equations of HNN were integrated with Adomian algorithm with a sub time step of $\delta =\frac{\tau }{200}$. These hyperparameters have been chosen as a trade-off between precision and the computational effort. Furthermore, different values of $\alpha$ were investigated. We found that another parameter that influences the performance of the network is the slope $\chi$ in Eq. (1). In particular, we selected $\chi =0.02$ for Caputo–Fabrizio definition and $\chi =4.0$ for Caputo definition of the fractional derivative.

In order to compare the performance of the algorithm, at different conditions (with $\alpha$ from 0.05 to 1.5), the mean squared error was calculated, that is, the average squared difference between the estimated values and the actual value:

30 $$\begin{aligned} MSE(\sigma )=\frac{1}{N}\sum _{k=1}^{N}(\hat{\sigma }_k-\sigma _k)^2 \end{aligned}$$

where $N=2000$ denotes the length of data used for parameter estimation, $\hat{\sigma }_k$ and $\sigma _k$ the estimated and actual parameter respectively.

With regard to fractional-order derivative, both Caputo–Fabrizio and Caputo equations were taken into account.

**Fig. 3:**
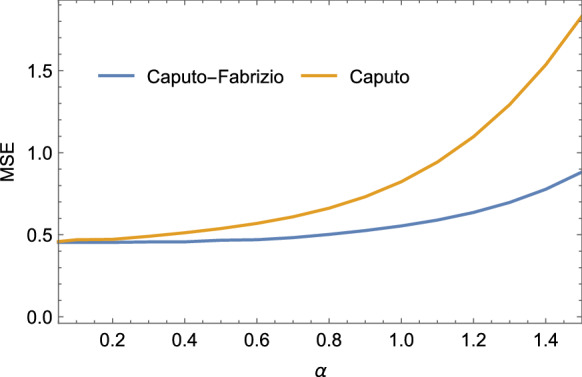
MSE for the $\sigma$ parameter estimation of the Lorenz system at different fractional-order values when the Caputo and Caputo–Fabrizio fractional-order derivatives are considered

**Fig. 4:**
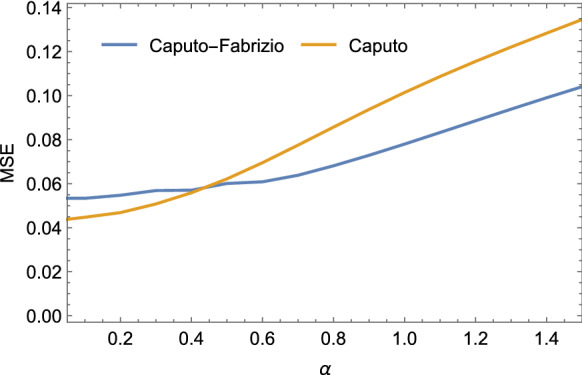
MSE obtained estimating the $\sigma , \rho$ and $\beta$ parameters of the Lorenz system as function of the fractional-order values

Figure 2a shows the results obtained for Caputo–Fabrizio FOHNN case, while the Caputo FOHNN case is reported in Fig. 2b. It can be noticed that, in both cases, fractional-order HNNs exhibit a better estimation capability, compared to the integer-order neuron structure when $\alpha <1$. Moreover, lower values of $\alpha$ provide a better estimation both in terms of precision and convergence time.

**Table 2:** The MSE obtained for parameters estimation in the Lorenz system with FOHNN for different values of $\alpha$ and applying the ant colony optimization as described in [26]

FO-HNN		
$\alpha$	MSE (C-F)	MSE (C)
0.05	$9.82\times 10^{-6}$	$5.71\times 10^{-6}$
0.1	$9.82\times 10^{-6}$	$6.06\times 10^{-6}$
0.2	$1.05\times 10^{-5}$	$6.91\times 10^{-6}$
0.3	$1.16\times 10^{-5}$	$8.63\times 10^{-6}$
0.4	$1.17\times 10^{-5}$	$1.11\times 10^{-5}$
0.5	$1.34\times 10^{-5}$	$1.47\times 10^{-5}$
0.6	$1.38\times 10^{-5}$	$2.02\times 10^{-5}$
0.7	$1.58\times 10^{-5}$	$2.84\times 10^{-5}$
0.8	$1.90\times 10^{-5}$	$3.90\times 10^{-5}$
0.9	$2.34\times 10^{-5}$	$5.17\times 10^{-5}$
1.0	$2.89\times 10^{-5}$	$6.59\times 10^{-5}$
1.1	$3.56\times 10^{-5}$	$8.10\times 10^{-5}$
1.2	$4.33\times 10^{-5}$	$9.66\times 10^{-5}$
1.3	$5.20\times 10^{-5}$	$1.13\times 10^{-4}$
1.4	$6.13\times 10^{-5}$	$1.29\times 10^{-4}$
1.5	$7.12\times 10^{-5}$	$1.47\times 10^{-4}$
PSO-ACO MSE: $1.20\times 10^{-5}$		

**Fig. 5:**
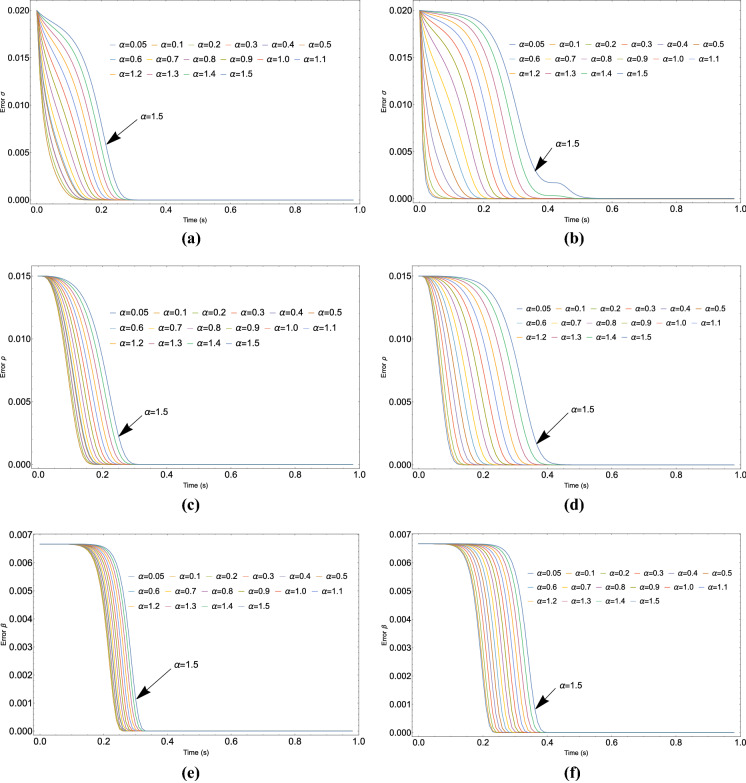
Estimation error of $\sigma$ (**a**, **b**), $\rho$ (**c**, **d**) and $\beta$ (**e**, **f**), of the Lorenz system adopting different values for the fractional-order parameter $\alpha$ in the case of Caputo–Fabrizio (left side) and Caputo (right side) derivatives

Table 1 and Fig. 3 report the estimation performance in term of MSE for each experiment.

The application of fractional-order systems improves the parameter estimation error when $\alpha <1$. Moreover, the reduction of the fractional parameter $\alpha$ is highly correlated with the improvement of the estimation performance. However, it can be noticed that for low values of $\alpha$ (i.e. $\alpha <0.4$) for the Caputo–Fabrizio case), the MSE reaches a plateau where further reductions of the fractional parameter value will produce a minimum impact on the estimation performance. In general, when $\alpha$ is very low (e.g. $\alpha <0.05$) numerical issues can arise during the simulations, requiring a further optimization of the adopted hyperparameters and an increase of the computational effort. The reported simulation results show better performance when Caputo–Fabrizio derivative is adopted although the estimation error converges to similar values when the parameter $\alpha$ is in the next to the bottom side of the considered range.

Our FOHNN architecture was also compared to the particle swarm optimization approach proposed in [26], where a hybrid swarm intelligence algorithm has been proposed for the estimation of $\sigma$, $\rho$ and $\beta$. In our experiments, an estimation of $\sigma$, $\rho$ and $\beta$ was conducted by using fractional-order HNN starting from an initial random estimation chosen in the following range:

31 $$\begin{aligned} \sigma \in [0,20], \quad \rho \in [0,50] \quad \text {and} \quad \beta \in [0,5] \end{aligned}$$

A total of nine experiments were conducted by varying $\alpha$ from 0.05 to 1.5. The Lorenz system was always integrated with $\tau =0.01$ for $N=100$ steps, while the FOHNN has been simulated with $\delta =\frac{\tau }{1000}$ at each time step.

We evaluated the accuracy of the identification process adopting the following index:

32 $$\begin{aligned}&\hbox {MSE}(\sigma , \rho , \beta )\nonumber \\&=\frac{1}{N}\sum _{k=1}^{N}(\hat{\sigma }_k-\sigma _k)^2+(\hat{\rho }_k-\rho _k)^2+(\hat{\beta }_k-\beta _k)^2\nonumber \\ \end{aligned}$$

Figure 4 shows the MSE as a function of $\alpha$ and the estimated parameters. Our algorithm outperforms the solution proposed in [26] terms of MSE as demonstrated in Table 2 when a fractional-order $\alpha \le$0.4 is considered. In this simulation, the Caputo–Fabrizio method slightly outperforms the Caputo one, for low values of $\alpha$ (i.e. $\alpha \le$0.4).

Further details are reported in Fig. 5 where the estimation error in the simultaneous searching of three unknown parameters $\sigma$, $\rho$ and $\beta$, using different values of $\alpha$ for both Caputo–Fabrizio and Caputo derivatives, is depicted. The obtained results

### Parameter estimation of a two-cart system

**Fig. 6:**
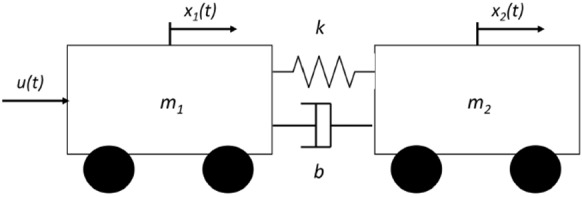
Model of a two-cart system with masses $m_1$ and $m_2$, connected with a spring–damping mechanism with two unknown parameters *k* and *b* and subject to an external force *u*(*t*)

In [4] HNNs have been studied for on-line parameter estimation and applied in a two-cart system connected with a spring-damping mechanism with two unknown parameters *k* and *b* (see Fig. 6).

In this simulation, the system under consideration is linear in parameters and then it can be written in the form $\varvec{y}=\varvec{A}(\varvec{x},\varvec{u})\varvec{\theta }$:

33 $$\begin{aligned} \varvec{y}&=\begin{pmatrix}m_1\frac{d^2}{dt^2}x_1(t) - u(t) \\ m_2\frac{d^2}{dt^2}x_2(t) \end{pmatrix}\nonumber \\ \varvec{A}&=\begin{pmatrix}-x_1(t)+x_2(t) &{} -\frac{d}{dt}x_1(t)+\frac{d}{dt}x_2(t) \\ x_1(t)-x_2(t) &{} \frac{d}{dt}x_1(t)-\frac{d}{dt}x_2(t) \end{pmatrix} \nonumber \\ \varvec{\theta }&= \begin{pmatrix}k \\ b \end{pmatrix} \end{aligned}$$

where $x_i$, $\frac{d}{dt}x_i(t)$, $\frac{d^2}{dt^2}x_i(t)$ and $m_i$ denote the displacement, velocity, acceleration and mass of cart *i*, respectively, and *u*(*t*) is the force applied to the cart 1. The unknown parameters to be identified are the spring constant *k* and the damper constant *b*.

In the following simulations, it was assumed that $m_1=m_2=2\ [\text {kg}]$, $k=1\ [\frac{\text {N}}{\mathrm {m}}]$, $b=0.1\ [\frac{\text {Ns}}{\mathrm {m}}]$ and two sets of initial conditions are considered:

- $$\text {IC}_1$$: $$x_i(0)=0\ [\text {m}]$$, $$\frac{d}{dt}\big |_{t=0}x_i(t)=0\ [\frac{\text {m}}{\mathrm {s}}]$$
- $$\text {IC}_2$$: $$x_i(0)=0\ [\text {m}]$$, $$\frac{d}{dt}\big |_{t=0}x_1(t)=1$$, $$\frac{d}{dt}\big |_{t=0}x_2(t)=2\ [\frac{\text {m}}{\mathrm {s}}]$$

Furthermore, two values of force applied to the cart 1 where considered: $u_1(t)=e^{-t}\ [\text {N}]$ and $u_2(t)=\text {sin}(\pi t)\ [\text {N}]$; initial estimates of *k* and *b* were randomly generated in the interval [0, 5] with uniform distribution.

**Fig. 7:**
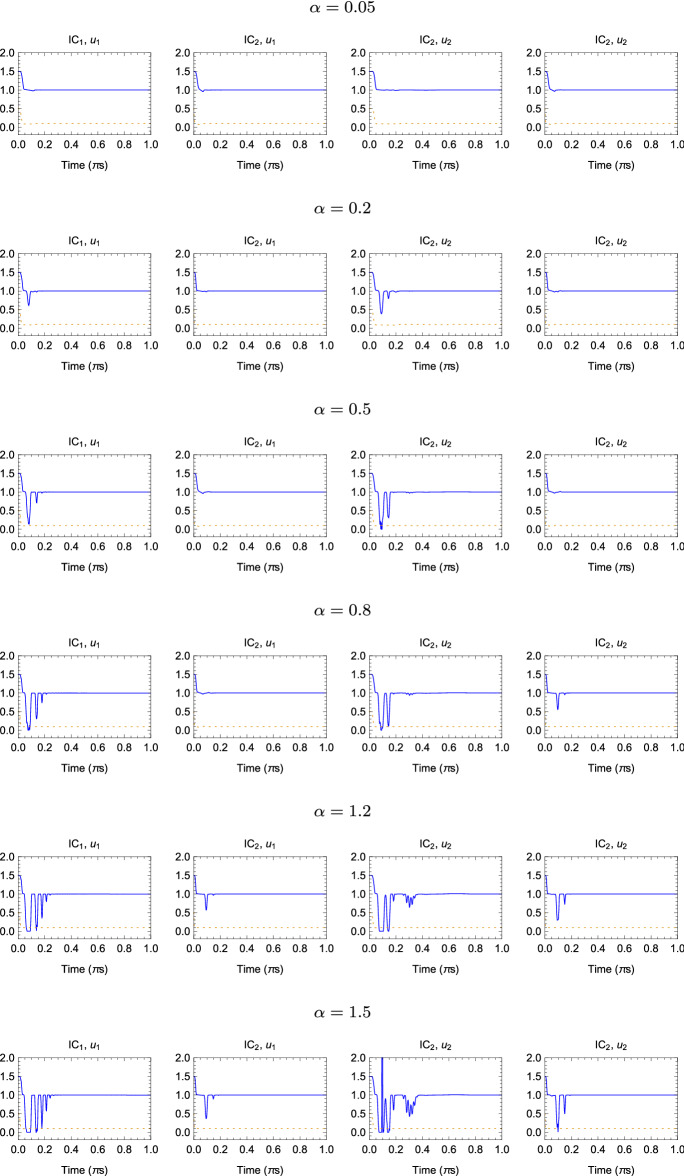
Time evolution of the parameter estimation obtained with the FOHNN using the Caputo–Fabrizio derivative, when $\alpha =[0.05,\; 0.2,\; 0.5,\; 0.8,\; 1.2,\; 1.5]$ and for different sets of initial condition IC and forces *u*, where $\text {IC}_1$: $x_i(0)=0\ [\text {m}]$, $\frac{d}{dt}\big |_{t=0}x_i(t)=0\ [\frac{\text {m}}{\mathrm {s}}]$, $\text {IC}_2$: $x_i(0)=0\ [\text {m}]$, $\frac{d}{dt}\big |_{t=0}x_1(t)=1$, $\frac{d}{dt}\big |_{t=0}x_2(t)=2\ [\frac{\text {m}}{\mathrm {s}}]$, $u_1(t)=e^{-t}\ [\text {N}]$ and $u_2(t)=\text {sin}(\pi t)\ [\text {N}]$. The solid and dashed lines represent the estimated values for parameters *k* and *b*, respectively

**Fig. 8:**
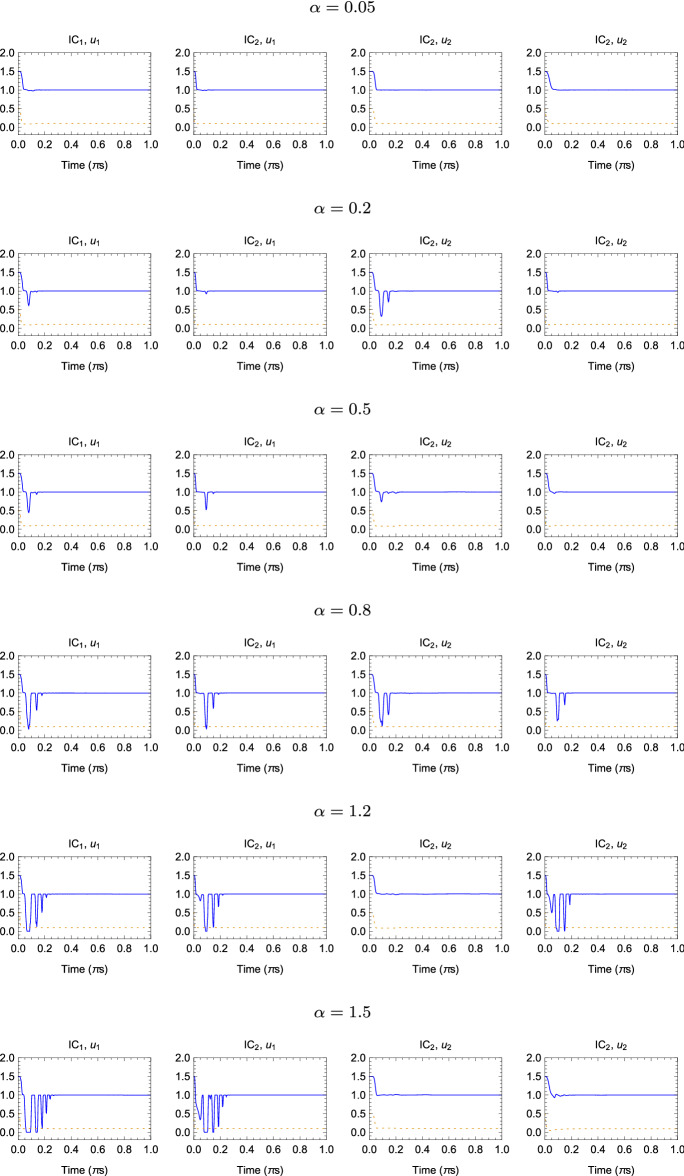
Time evolution of the parameter estimation obtained with the FOHNN using the Caputo derivative, when $\alpha =[0.05,\; 0.2,\; 0.5,\; 0.8,\; 1.2,\; 1.5]$ and for different sets of initial condition IC and forces *u*. The solid and dashed lines represent the estimated values for parameters *k* and *b*, respectively

The time evolution of the estimated parameters is represented in Fig. 7 for the Caputo–Fabrizio derivative and in Fig. 8 for Caputo derivative. The FOHNN behaviour is evaluated for a subset of $\alpha$ values extract from the considered range. The entire sets of combinations, including initial conditions IC and forces *u*(*t*), are reported. All simulations have been performed with the following hyperparameters: $\tau =0.01$ and $\delta =\frac{\tau }{200}$.

In order to evaluate the improvement of using different fractional-order $\alpha$, the estimation error (ER) at time *t* was calculated, defined as:

34 $$\begin{aligned} \text {ER(t)}=\sqrt{(k-\hat{k})^2+(b-\hat{b})^2} \end{aligned}$$

**Table 3:** Settling times ($\pi$s) at which the estimation error ER is below $5\times 10^{-3}$ for the two-cart system parameter identification in different operational conditions when the Caputo–Fabrizio derivative is considered

$\alpha$	$\text {IC}_1$, $u_1$	$\text {IC}_2$, $u_1$	$\text {IC}_1$, $u_2$	$\text {IC}_2$, $u_2$
0.05	$1.30\times 10^{-1}$	$8.30\times 10^{-2}$	$2.30\times 10^{-1}$	$8.60\times 10^{-2}$
0.1	$1.30\times 10^{-1}$	$1.00\times 10^{-1}$	$2.30\times 10^{-1}$	$1.1\times 10^{-1}$
0.2	$1.50\times 10^{-1}$	$1.10\times 10^{-1}$	$2.30\times 10^{-1}$	$1.2\times 10^{-1}$
0.3	$1.70\times 10^{-1}$	$1.10\times 10^{-1}$	$3.10\times 10^{-1}$	$1.3\times 10^{-1}$
0.4	$1.90\times 10^{-1}$	$1.10\times 10^{-1}$	$3.30\times 10^{-1}$	$1.3\times 10^{-1}$
0.5	$1.90\times 10^{-1}$	$1.20\times 10^{-1}$	$3.50\times 10^{-1}$	$1.3\times 10^{-1}$
0.6	$2.20\times 10^{-1}$	$1.20\times 10^{-1}$	$4.50\times 10^{-1}$	$1.6\times 10^{-1}$
0.7	$2.20\times 10^{-1}$	$1.20\times 10^{-1}$	$4.50\times 10^{-1}$	$1.6\times 10^{-1}$
0.8	$2.20\times 10^{-1}$	$1.20\times 10^{-1}$	1.00	$1.60\times 10^{-1}$
0.9	$2.50\times 10^{-1}$	$1.60\times 10^{-1}$	1.00	$1.60\times 10^{-1}$
1.0	$2.50\times 10^{-1}$	$1.60\times 10^{-1}$	1.00	$1.60\times 10^{-1}$
1.1	$2.50\times 10^{-1}$	$1.60\times 10^{-1}$	1.00	$1.60\times 10^{-1}$
1.2	$2.70\times 10^{-1}$	$1.60\times 10^{-1}$	1.00	$1.60\times 10^{-1}$
1.3	$2.70\times 10^{-1}$	$1.70\times 10^{-1}$	1.00	$1.90\times 10^{-1}$
1.4	$2.70\times 10^{-1}$	$1.70\times 10^{-1}$	1.00	$1.90\times 10^{-1}$
1.5	$2.70\times 10^{-1}$	$1.90\times 10^{-1}$	1.00	$1.90\times 10^{-1}$

**Table 4:** Times ($\pi$s) at which the estimation error ER is below $5\times 10^{-3}$ for the two-cart system parameter identification in different operational conditions when the Caputo derivative is considered

$\alpha$	$\text {IC}_1$, $u_1$	$\text {IC}_2$, $u_1$	$\text {IC}_1$, $u_2$	$\text {IC}_2$, $u_2$
0.05	$1.30\times 10^{-1}$	$1.10\times 10^{-1}$	$6.00\times 10^{-2}$	$9.20\times 10^{-2}$
0.1	$1.50\times 10^{-1}$	$1.10\times 10^{-1}$	$6.00\times 10^{-2}$	$1.10\times 10^{-1}$
0.2	$1.50\times 10^{-1}$	$1.10\times 10^{-1}$	$2.10\times 10^{-1}$	$1.10\times 10^{-1}$
0.3	$1.50\times 10^{-1}$	$1.60\times 10^{-1}$	$2.30\times 10^{-1}$	$1.20\times 10^{-1}$
0.4	$1.70\times 10^{-1}$	$1.60\times 10^{-1}$	$2.30\times 10^{-1}$	$1.20\times 10^{-1}$
0.5	$1.80\times 10^{-1}$	$1.70\times 10^{-1}$	$2.30\times 10^{-1}$	$1.20\times 10^{-1}$
0.6	$1.90\times 10^{-1}$	$1.70\times 10^{-1}$	$2.30\times 10^{-1}$	$1.30\times 10^{-1}$
0.7	$1.90\times 10^{-1}$	$1.90\times 10^{-1}$	$2.30\times 10^{-1}$	$1.60\times 10^{-1}$
0.8	$2.20\times 10^{-1}$	$1.90\times 10^{-1}$	$3.30\times 10^{-1}$	$1.60\times 10^{-1}$
0.9	$2.20\times 10^{-1}$	$1.90\times 10^{-1}$	$4.40\times 10^{-1}$	$1.60\times 10^{-1}$
1.0	$2.20\times 10^{-1}$	$2.30\times 10^{-1}$	$4.40\times 10^{-1}$	$1.60\times 10^{-1}$
1.1	$2.50\times 10^{-1}$	$2.30\times 10^{-1}$	$4.50\times 10^{-1}$	$1.60\times 10^{-1}$
1.2	$2.50\times 10^{-1}$	$2.30\times 10^{-1}$	$4.50\times 10^{-1}$	$2.90\times 10^{-1}$
1.3	$2.70\times 10^{-1}$	$2.50\times 10^{-1}$	$4.50\times 10^{-1}$	$2.90\times 10^{-1}$
1.4	$2.70\times 10^{-1}$	$2.50\times 10^{-1}$	$4.50\times 10^{-1}$	$4.40\times 10^{-1}$
1.5	$3.00\times 10^{-1}$	$2.50\times 10^{-1}$	$4.50\times 10^{-1}$	$5.70\times 10^{-1}$

Tables 3 and 4 show the settling times (in $\pi$s) at which $\text {ER}<5\times 10^{-3}$ for each experiment considering both Caputo–Fabrizio and Caputo definitions of the fractional-order derivative. Also in this case, low values of $\alpha$ correspond to better performance if compared with the integer-order case reported in [4]. The effect of the derivate definition is different among the considered cases: in the first and fourth set-ups ($\text {IC}_1$, $u_1$) and ($\text {IC}_2$, $u_2$), similar results are obtained in particular for low values of the parameter $\alpha$; in the second set-up ($\text {IC}_1$, $u_2$), the Caputo–Fabrizio method outperforms the Caputo solution regardless of the selected $\alpha$; and in the third set-up ($\text {IC}_2$, $u_1$), the results obtained adopting the Caputo definition outperform the Caputo–Fabrizio solution.

## Conclusions

In this work, an application of fractional-order Hopfield neural network was investigated for on-line parameters estimation of nonlinear dynamical models. In particular, it was found in the simulations that fractional order influences the convergence of the parameter estimation process. The selection of the Fractional-order derivative definition is another important aspect to investigate. Furthermore, simulations have been performed using Adomian decomposition method which has been confirmed as a reliable algorithm for solving fractional-order differential equations.

As known in the literature for integer-order neurons, also in the proposed approach the main requirements for the application in parameter estimation are that parameters must have a limited known variation range and that dynamical system equations must be linear in parameters. It was demonstrated for two different cases of study that the proposed approach can outperform other methods available in literature exploiting the properties of fractional-order systems that are better responsive than the integer-order one and are able to capture complex behaviours, such as the long-term memory effects of the dynamics. In particular, the fractional-order parameter $\alpha$ represents a key element to be selected. The selection of the parameter $\alpha >1$ does not lead to good results, whereas, for $\alpha <1$, the estimation of the parameters improves both in terms of convergence time and accuracy. It is important to note that often the improvements obtained tend to stabilize once an optimal alpha value is reached. However, this value depends on the specific case of study investigated. It seems to be unnecessary to assign very small values to the parameter $\alpha$ (i.e. below the range considered). However, this action would require further optimization of the hyperparameters to avoid numerical problems. The choice of the fractional-order derivative definition is a further element to be considered for the optimization of the FOHNN. The results obtained show that when $\alpha$ is close to the top of the considered range the Caputo–Fabrizio derivative is more efficient than the Caputo definition, while, in the bottom of the considered $\alpha$ range, the two approaches are either equivalent or there is a specific preference based on the case study under consideration. Further works will include the application of the proposed architecture in adaptive control schemes analysing the stability of the related closed-loop systems.
